# Prediction of CYP-Mediated Drug Interaction Using Physiologically Based Pharmacokinetic Modeling: A Case Study of Salbutamol and Fluvoxamine

**DOI:** 10.3390/pharmaceutics15061586

**Published:** 2023-05-24

**Authors:** Lara Marques, Nuno Vale

**Affiliations:** 1 OncoPharma Research Group, Center for Health Technology and Services Research (CINTESIS), Rua Doutor Plácido da Costa, 4200-450 Porto, Portugal; lara.marques2010@hotmail.com; 2 Faculty of Medicine, University of Coimbra, Azinhaga de Santa Comba, Celas, 3000-548 Coimbra, Portugal; 3 CINTESIS@RISE, Faculty of Medicine, University of Porto, Al. Prof. Hernâni Monteiro, 4200-319 Porto, Portugal; 4 Department of Community Medicine, Health Information and Decision (MEDCIDS), Faculty of Medicine, University of Porto, Rua Dr. Plácido da Costa, 4200-450 Porto, Portugal

**Keywords:** drug–drug interaction, salbutamol, fluvoxamine, PBPK modeling, pharmacokinetics, simulation, CYP-mediated metabolism

## Abstract

Drug–drug interactions (DDIs) represent a significant concern in healthcare, particularly for patients undergoing polytherapy. DDIs can lead to a range of outcomes, from decreased therapeutic effectiveness to adverse effects. Salbutamol, a bronchodilator recommended for the treatment of respiratory diseases, is metabolized by cytochrome P450 (CYP) enzymes, which can be inhibited or induced by co-administered drugs. Studying DDIs involving salbutamol is crucial for optimizing drug therapy and preventing adverse outcomes. Here, we aimed to investigate CYP-mediated DDIs between salbutamol and fluvoxamine through in silico approaches. The physiologically based pharmacokinetic (PBPK) model of salbutamol was developed and validated using available clinical PK data, whereas the PBPK model of fluvoxamine was previously verified by GastroPlus. Salbutamol–fluvoxamine interaction was simulated according to different regimens and patient’s characteristics (age and physiological status). The results demonstrated that co-administering salbutamol with fluvoxamine enhanced salbutamol exposure in certain situations, especially when fluvoxamine dosage increased. To sum up, this study demonstrated the utility of PBPK modeling in predicting CYP-mediated DDIs, making it a pioneer in PK DDI research. Furthermore, this study provided insights into the relevance of regular monitoring of patients taking multiple medications, regardless of their characteristics, to prevent adverse outcomes and for the optimization of the therapeutic regimen, in cases where the therapeutic benefit is no longer experienced.

## 1. Introduction

Diseases demanding combination therapy or patients with a broad spectrum of diseases involve a regimen of two or more drugs. Taking multiple medications simultaneously is the key driver for the increased risk of undesirable drug–drug interactions (DDIs). These interactions may lead to altered drug profiles, raise the likelihood of adverse reactions (ADRs), and ultimately, life-threatening outcomes [1,2]. Currently, DDIs represent a global burden during pharmacological therapy. It is estimated that about 10% of people take five or more drugs concomitantly, and this number soars among elderly people [3]. For this reason, clinicians face two hurdles as far as DDIs are concerned. First, the selection of drugs that can be administered together safely and effectively ensures a high-quality treatment, which is increasingly challenging and complex. A posteriori, in the management of DDIs, to reverse potential ADRs, resorting to strategies including drug dosage adjustments, drug level monitoring, and alternative medications or routes of administration use may be necessary [4].

The conceptual framework of a DDI is divided into pharmacokinetic (PK) and pharmacodynamic (PD), resulting in an altered free plasma drug concentration or in the intensification or antagonism of the clinical effect, respectively. Both interactions might cause either a decrease in therapeutic effectiveness or an increase in toxicity [5,6,7,8]. Drug-metabolizing enzymes and drug transporters are the epicenters of PK-mediated DDI studies. The monooxygenases metabolizing enzymes, also known as the cytochrome P450 (CYP) superfamily, are involved in the phase I metabolism of approximately 45% of marketed drugs. These enzymes have numerous isoforms, of which the most relevant for DDI studies are the 1A2, 3A4/5, 2B6, 2C9, 2C19, and 2D6 [9]. PK interactions, as stated, result from changes in CYP-mediated metabolism through the inhibition or induction of their enzyme expression [5,10]. Inhibiting CYP will influence PK parameters, such as the maximum concentration (C_max_) and area under curve (AUC), reflected in the increased drug bioavailability [11]. These variations may have positive outcomes, such as greater therapeutic effectiveness, or negative consequences if toxicity is enhanced. The induction of CYP, on the other hand, promotes the opposite effect.

Salbutamol, a short-acting β_2_-agonist (SABA) recommended for asthma treatment, is among the multiple drugs used in polytherapy. According to the latest Global Initiative for Asthma (GINA) guidelines, salbutamol monotherapy is contraindicated [12]. Thus, this SABA is frequently combined with other medicines, especially inhaled corticosteroids (ICS) and other bronchodilators [13]. In addition, due to the high prevalence of this chronic disease, there are several disorders that may coexist with asthma [14]. For this reason, asthma sufferers usually undergo polypharmacy.

**Figure 1:**
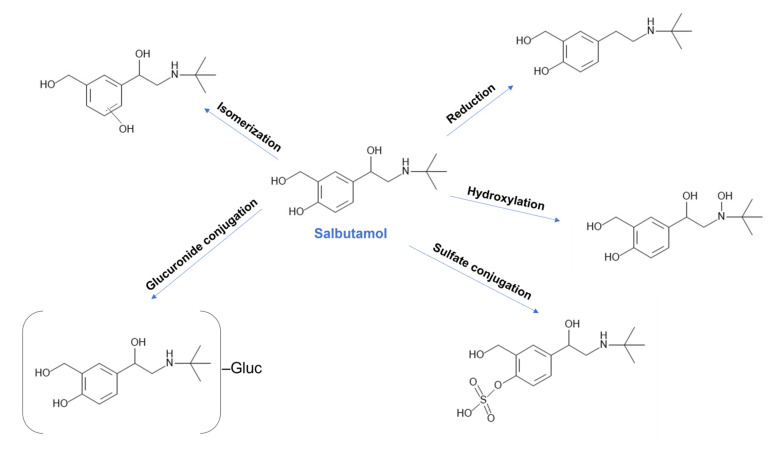
Main metabolic pathways of salbutamol. Created with MedChem Designer.

Therefore, investigating the effect of co-administered drugs on salbutamol is relevant to improving the efficacy and safety of this drug. Having in-depth knowledge of salbutamol’s metabolic profile is essential for this sort of study. Salbutamol metabolism includes several pathways (Figure 1) [15]. The main route of metabolism is sulfate conjugation, where sulfotransferase enzymes are involved. Nevertheless, due to the swallowing effect, and in cases of oral administration, the CYP450 enzyme system is recognized as playing an active role in the hepatic metabolism of salbutamol [16,17,18].

**Figure 2:**
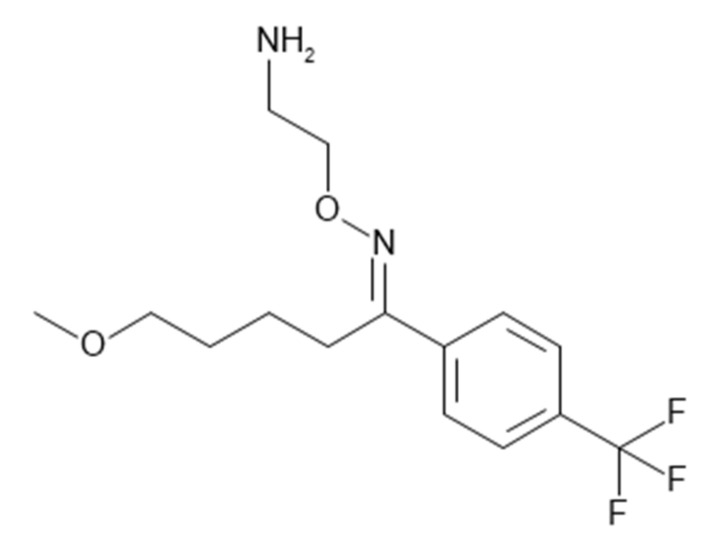
Chemical structure of drug fluvoxamine. Created with MedChem Designer.

As previously stated [19], despite drug agencies’ attempts to explore PK DDIs (driven in part by the inherent difficulty of investigating PD DDIs), this type of research on bronchodilator medicines, namely salbutamol, is quite limited. In silico or computer-based simulation software then emerges as an important tool for improving drug characterization when extensive preclinical and clinical data are scarce. Indeed, salbutamol is often combined with other drugs, either other bronchodilators or drugs that treat other concomitant conditions. Herein, we aimed to predict potential CYP-mediated DDI between salbutamol and fluvoxamine (Figure 2), through in silico approaches. To this end, in a preliminary phase, several potential interactor drugs were screened, with fluvoxamine, an antidepressant, being the eligible candidate. Subsequently, physiologically based pharmacokinetic (PBPK) models that mimic different patient characteristics (age, renal function, pregnancy, weight) were developed for both salbutamol and fluvoxamine and a DDI simulation was conducted.

## 2. Materials and Methods

### 2.1. Prediction of Pharmacokinetic and Physicochemical Properties of Salbutamol

Salbutamol was characterized according to its physicochemical and PK properties using ADMET Predictor^®^ (Version 10.4; Simulation Plus Inc., Lancaster, CA, USA), a software tool that accurately predicts several features of compounds, including physicochemical and PK properties. The chemical structure of salbutamol was drawn in MedChem Designer (Version 5.5; Simulation Plus Inc., Lancaster, CA, USA) and then imported into ADMET Predictor^®^ in an MOL file format. Parameters such as Log P, molecular weight, solubility, human jejunum effective permeability (Peff), diffusion coefficient (Diff. Coeff.), CYP-mediated metabolism and transport, and blood–brain barrier (BBB) permeability were estimated using this software tool.

The PKs of salbutamol were simulated with a 4 mg dose given orally over 24 h, using an ADMET Predictor^®^ functionality (%Fa and %Fb calculator).

### 2.2. Screening of Potential Drug Interactors with Salbutamol

The screening of the drugs was performed using ADMET Predictor^®^. A preliminary analysis of drugs frequently combined with salbutamol was conducted. Corticosteroids, anticholinergics, beta-blockers, and others were included in this pre-selection of potential interactors (Supplementary Materials Table S1).

The MOL file of each drug, obtained with MedChem Designer, was uploaded into the program. All absorption, distribution, metabolism, excretion, and toxicity (ADMET) properties were then predicted, particularly the metabolism mediated by CYP enzymes (CYP1A2, CYP2A6, CYP2B6, CYP2C8, CYP2C9, CYP2C19, CYP2D6, CYP2E1, and CYP3A4). These determined characteristics were subsequently compared to the predictions derived for salbutamol. The selection of the perpetrator drug was based on the similarity between its metabolic profile and that of salbutamol.

### 2.3. PBPK Modeling Development

The PBPK models for salbutamol were developed using GastroPlus software (Version 9.8.3; Simulation Plus Inc., Lancaster, CA, USA). The chemical structure of salbutamol and all the physicochemical and PK parameters previously computed by ADMET Predictor^®^ were imported into this software. Therefore, except for the «Gut Physiology» tab, where we specified the individual characteristics, all sections of the program used predicted values. 

The PK parameters of salbutamol were simulated with a 4 mg dose administered orally every 6 h. Observed values of bioavailability (Fa, fraction absorbed; FDp, fraction of the drug concentration in the portal vein; and F, fraction of the drug concentration in blood), maximum plasma concentration (C_max_), time required to maximum plasma concentration (T_max_), area under the curve (AUC), and maximum concentration in the liver (C_maxLiver_), were derived from ADMET Predictor^®^. The drug disposition-based parameters were determined in a compartmental PK model in a virtual 30-year-old healthy American male patient. The simulation duration was 24 h and provided quantitative and visual (plots) outputs of the PK features. The American population represents a large proportion of salbutamol users, as the estimated number of prescriptions in the United States was 61 million [20]. Therefore, our PBPK models included this reference group.

Several characteristics of the subjects, namely age, weight, and health status were modeled for the DDI simulations. Subjects aged 10, 30, and 65 years were included. Weight was established according to the body mass index (BMI) scale, where a BMI of 18.5–24.9 is normal, a BMI of 25–29.9 is overweight, and a BMI ≥ 30 is obese. The health status evaluated in this study was the different severities of renal impairment (mild, moderate, and severe) based on the estimated glomerular filtration rate (eGFR). Healthy was stated as not having any renal or hepatic impairment or weight issues. Additionally, we developed two PBPK models of a healthy woman and a healthy 10-week-pregnant woman. Detailed characteristics of these individuals are summarized in Supplementary Table S2.

### 2.4. PBPK Model Validation

The PK parameter values obtained from the developed models were compared with the literature data. Additionally, a visual inspection of the plots of the plasma concentration profile was performed to establish confidence between the PBPK models and similar studies reported in the literature. The PBPK models were therefore validated.

### 2.5. DDI Simulation between Salbutamol and Fluvoxamine

After selecting the eligible co-administered drug (fluvoxamine), DDI for salbutamol and fluvoxamine was conducted using the dynamic simulation and the steady-state mode in the DDI module of GastroPlus. The previously computed dataset was employed as input for the DDI prediction, considering the inhibitory effect of fluvoxamine as a perpetrator on salbutamol (victim). In turn, the inhibition enzyme kinetics constants (Ki, IC_50_) and induction kinetics constant (EC_50_) of the perpetrator were already integrated into the software, since the PBPK model of fluvoxamine had been validated by GastroPlus. 

The simulations were run according to the previously developed PBPK models. Firstly, the interaction of fluvoxamine on salbutamol was predicted using the PBPK model of a healthy 30-year-old man for 24 h in both dynamic and steady-state modes. Three dose regimens were simulated: 100, 200, and 300 mg, one tablet per day. These doses were obtained from the literature. Subsequently, we used the other PBPK models (different ages and comorbidities) to investigate the DDI of fluvoxamine and salbutamol under different conditions. These predictions were conducted in steady-state mode. 

The classification of DDI is based on the AUC ratio in the presence or absence of the perpetrator and is categorized as no interaction, weak, moderate, or strong. With an AUC ratio between 1.25 and 2, the interaction is weak. A moderate interaction is defined with an AUC ratio range between 2 and 5. When the AUC ratio > 5, the interaction is considered to be strong.

## 3. Results and Discussion

Salbutamol has been combined with several drugs, as salbutamol monotherapy is contraindicated [12]. Furthermore, the high prevalence of respiratory diseases worldwide, particularly asthma, leads to the occurrence of comorbidities, or coexisting diseases, requiring the prescription of more than one drug [14]. Many clinical studies have reported the polytherapy-associated increased risk of DDIs. By contrast, little is known about salbutamol PK DDIs. In order to study, for the first time, the PK interaction between salbutamol and fluvoxamine, the physicochemical properties of salbutamol were estimated by ADMET Predictor^®^ (Table 1). The different attributes were compared to values of the main drug databases and with values obtained from other predictive platforms for ADME properties, namely SwissADME and pkCSM (optimized values). As noted, the accuracy of the simulated data is rather considerable, allowing us to proceed with this study. 

### 3.1. Fluvoxamine as the Perpetrator Drug for Salbutamol DDI Study

The metabolic profile of salbutamol was examined using ADMET Predictor^®^. Therefore, the phase I metabolic reactions rely on the participation of the enzymes CYP2C19 and CYP2D6, as outlined in Table 2. Among the 9 CYP superfamily enzymes included in this computer software, salbutamol is a substrate of CYP2C19 and CYP2D6, with an accuracy of 66% and 82%, respectively. This prediction also suggests that this bronchodilator is, with a 49% likelihood, a CYP2D6 inhibitor. In addition to the metabolization sites, data for the enzyme’s affinity for the substrate (Km, Michaelis–Menten constant), maximum rate of metabolization (V_max_), and intrinsic clearance (CL) are also provided.

The spectrum of drugs that may be co-administered with salbutamol is extensive, ranging from beta-blockers for heart diseases to antidepressants [19]. The screening of drugs for potential interactions with salbutamol included 17 compounds, whose ADMET properties were predicted (Supplementary Materials Table S3). The drug selection for our study was based on the analysis of the CYP-metabolizing enzymes of each drug. Since the GastroPlus DDI module is exclusively focused on CYP enzyme-mediated interactions, we established the criterion of electing the perpetrator for having at least one salbutamol-metabolizing CYP enzyme. Therefore, fluvoxamine was selected, being metabolized by CYP2C19 and CYP2D6 (Table 2). The respective prediction probabilities point to 67% and 66%. Our screening identified, in addition to fluvoxamine, other equally relevant drugs. Detailed information about these drugs’ metabolism is displayed in the Supplementary Materials (Table S3). Nonetheless, due to the lack of clinical data for required software inputs, we chose fluvoxamine for our DDI study, as it is a GastroPlus-verified model.

Fluvoxamine (Figure 2) is a selective serotonin reuptake inhibitor (SSRI) and a sigma-1 receptor agonist, recommended for the treatment of depression and other psychological conditions [25]. Interestingly, this antidepressant received a lot of attention during the pandemic. Several studies have demonstrated benefits of using fluvoxamine in the treatment of patients with COVID-19 [26,27,28]. Therefore, and because asthmatics constitute a risk group for the COVID-19 infection, the co-administration of salbutamol and fluvoxamine is likely to occur. Additionally, as we previously stated, the high prevalence of asthma means that there are many asthmatics with other coexisting diseases, and therefore it is likely that an asthmatic patient has a psychological condition that requires treatment with fluvoxamine.

Our prediction of metabolic properties defined fluvoxamine as an inhibitor of CYP2D6 and CYP3A4, with a likelihood of 70% and 80%, respectively. In addition to being a substrate for CYP2C19 and CYP2D6, it is also metabolized by CYP1A2 (albeit its prediction likelihood is low) and by CYP2E1. Several studies have reported the interference of fluvoxamine in the metabolism of other drugs via CYP2D6, CYP2C19, and CYP1A2 inhibition [29,30,31,32]. Our findings, however, indicate that fluvoxamine is a CYP2C19 inhibitor with a likelihood of 95% and 51% of being a CYP1A2 inhibitor. Undoubtedly, our results contradict the existing literature regarding CYP2D6. In vitro studies with liver microsomes should be conducted to support our data. Notwithstanding, since salbutamol is also metabolized by CYP2D6, the interaction between these two compounds may occur through this pathway. However, we should not rule out the influence that fluvoxamine may have on salbutamol in terms of other pathways, namely because both are CYP2D6 substrates and inhibitors.

### 3.2. PBPK Model for Salbutamol

**Figure 3:**
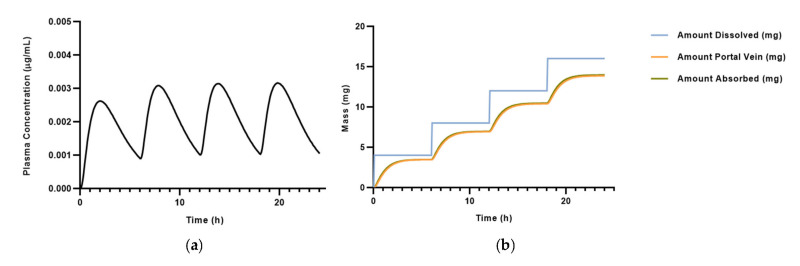
Pharmacokinetics of 4 mg q6h salbutamol over 24 h simulation in a 30-year-old American male: (**a**) evaluation of salbutamol plasma concentration over time, and (**b**) amount of drug in the portal vein, absorbed, and dissolved over time.

The PK properties were first estimated by ADMET Predictor^®^ and then transposed to GastroPlus (Table 3). Some of these characteristics (FDp, F, and C_max liver_) were not defined owing to the limitations of ADMET Predictor^®^. The «Pharmacokinetics» function computed the PK parameters in a healthy 30-year-old American male treated with 4 mg q6h (every 6 h) oral salbutamol, as detailed in the experimental procedures section. These values were also confirmed according to the literature [23]. According to Morgan et al. [33], peak oral salbutamol concentrations varied between 10.0 and 16.9 ng/mL and occurred from 1.0–4.0 h after the administration. Figure 3 illustrates the salbutamol systemic distribution in the defined PBPK model over a 24 h simulation.

### 3.3. Effect of Different Doses of Fluvoxamine on Salbutamol Pharmacokinetics

Aiming to examine the salbutamol–fluvoxamine interaction under different conditions, first, we modelled three different doses of fluvoxamine (100, 200, and 300 mg SID, once daily) on salbutamol kinetics in a healthy 30-year-old American male undergoing fixed-dose salbutamol therapy (4 mg every 6 h). In this study, we assumed that CYP2C19 and CYP2D6 are the exclusive enzymes of salbutamol metabolism (about 23% of the drug is metabolized by other enzymes) and therefore we investigated the fluvoxamine’s inhibitory effect on these enzymes.

**Figure 4:**
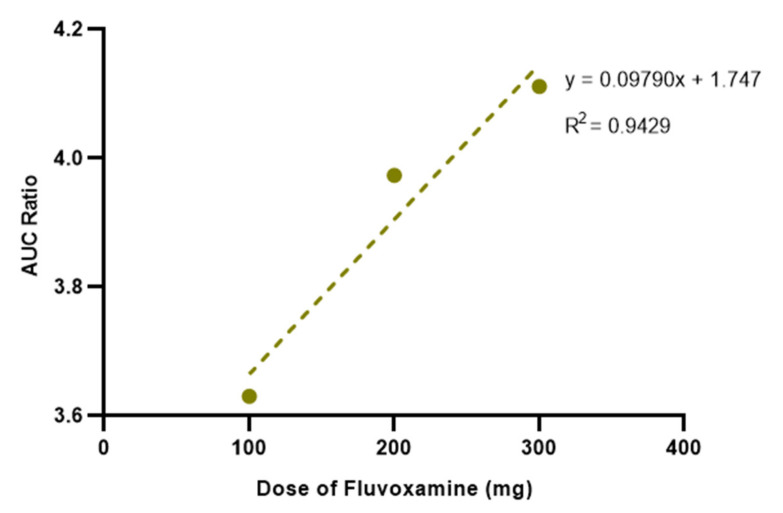
Effect of increasing fluvoxamine dose on the AUC ratio of salbutamol estimated by steady-state prediction in a 30-year-old American male undergoing fixed-dose salbutamol (4 mg q6h) and SID fluvoxamine therapy.

The interaction between fluvoxamine and salbutamol was first simulated in the steady-state mode. Figure 4 depicts the interaction-derived-AUC ratio as a function of fluvoxamine dosage. For a dose of 100 mg, an AUC ratio of 3.630 was recorded, indicating a moderate interaction. When salbutamol is co-administered with 200 mg of fluvoxamine, the AUC ratio increases to 3.973. The highest dose (300 mg) corresponds to an AUC ratio of 4.111. Therefore, a proportional increase in the AUC ratio is observed as the dose of fluvoxamine increases.

Thereafter, the PK parameters of each combination (salbutamol 4 mg + fluvoxamine 100 mg, salbutamol 4 mg + fluvoxamine 200 mg, and salbutamol 4 mg + fluvoxamine 300 mg) were compared to the salbutamol baseline (administered alone), through dynamic simulation (Table 4). The administration of 100 mg fluvoxamine (usual effective dose) with 4 mg salbutamol (recommended oral dose), despite the barely noticeable variations, resulted in an increase in all parameters except Fa, FDp, and T_max_. Fa and FDp refer to the drug bioavailability which, as expected, decreased (not significantly). In turn, the T_max_ of combined therapy was twice as low as the T_max_ of baseline salbutamol. These results support the literature. Fluvoxamine may decrease the clearance of salbutamol, contributing to increased salbutamol serum levels [34]. Hence, if fluvoxamine is effectively a CYP2D6 inhibitor, we can easily hypothesize that this inhibition may reduce the rate of salbutamol metabolism via CYP2D6. 

**Figure 5:**
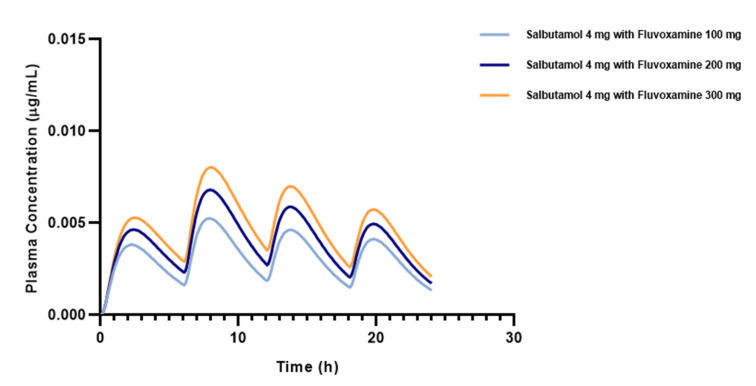
Effect of increasing fluvoxamine dose on the salbutamol plasma concentration estimated by dynamic simulation in a 30-year-old American male undergoing fixed-dose salbutamol (4 mg q6h) and SID fluvoxamine therapy.

Co-administration of salbutamol with higher doses of fluvoxamine resulted in a proportional increase in all PK parameters except Fa, FDp, and T_max_, which remained practically constant. In detail, increasing the fluvoxamine dosage does not influence salbutamol absorption, suggesting that such interaction likely occurs at the metabolism level. The drug fraction measured in the portal vein (FDp), in turn, increases significantly when salbutamol is combined with 200 and 300 mg of fluvoxamine, in contrast to the most frequent combination (salbutamol 4 mg + fluvoxamine 100 mg). The portal vein, being the site of drug entrance into the hepatic systemic where metabolism takes place, displays similar drug concentration values. As before, this metabolism-unrelated parameter is not changed. We highlight that these parameters may not be accurately predicted, because this software is based on metabolism-mediated interactions. In addition, our findings demonstrate a decrease in the proportion of salbutamol in the bloodstream as the dose of fluvoxamine increases. As a CYP2D6 inhibitor, fluvoxamine may impact salbutamol’s metabolism rate through enzyme inhibition, leading to a higher plasma concentration (Figure 5). As a result of the greater salbutamol non-metabolized fraction, the AUC and C_max_ values are likewise increased. From this standpoint, our results underline the need for monitoring in cases when fluvoxamine ought to be given in higher dosages to asthmatic patients undergoing salbutamol treatment, as the risk of toxicity and ADRs increases. 

### 3.4. Effect of Different Ages on Salbutamol Pharmacokinetics Co-Administered with Fluvoxamine

**Figure 6:**
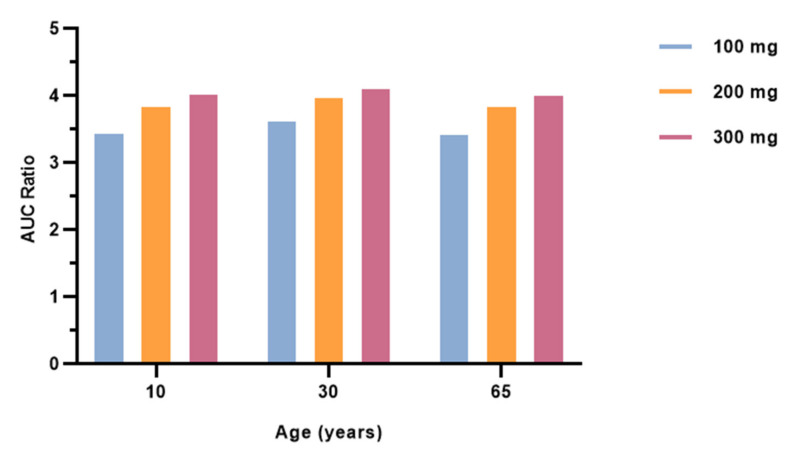
Effect of increasing fluvoxamine dose on the AUC ratio of salbutamol estimated by steady-state prediction in 10-, 30-, and 65-year-old virtual subjects undergoing fixed-dose salbutamol (4 mg q6h) and SID fluvoxamine therapy.

To investigate whether age had an influence on the co-administration of fluvoxamine and salbutamol, we simulated this therapeutic regimen in virtual male American subjects aged 10, 30, and 65 years (Table 5, Figure 6). In fact, age has been identified as the cornerstone of hepatic clearance alterations, since the rate of drug metabolism in the liver depends on its capacity to remove the drug from systemic circulation, as well as drug uptake into hepatocytes and enzyme activity, parameters that change over time [35,36,37]. For instance, children metabolize medications faster than adults. Although the underlying cause of this phenomenon is unclear, the increased ratio of liver size to body size in children is thought to be the main driver of increased enzyme activity. In addition, CYP450 enzymes have different expression levels depending on age [36,37,38,39]. Some are active during pregnancy, while others fully develop days, months, or even years after birth. In the context of our study, CYP2C19 expression reaches adult levels around 10 years of age, whereas CYP2D6 enzyme activity reaches the average adult activity after 5 years of age. At earlier ages, CYP activity exceeds adult levels [39]. Keeping this in mind, we are unable to draw correlations from our results, since the simulated pediatric age was 10 years. Further studies should be conducted to understand whether the administration of more than one drug influences CYP metabolism in younger individuals with enhanced CYP activities. Notwithstanding, our findings reveal that salbutamol concentrations are reduced in 10-year-old children, suggesting that salbutamol–fluvoxamine interaction has no enhanced impact at this age. Of note, the AUC ratio of the liver concentrations unbound, defined as the true inhibitor concentration that determines CYP-mediated DDI, was significantly increased in the PBPK model that mimics pediatric age, compared with adult age.

In elderly people, drug metabolism may be delayed due to altered CYP enzyme function, and reduced liver mass and blood flow. In fact, several animal studies have documented age-related changes in CYP levels, despite human research failing to demonstrate such a correlation [35,40,41]. Investigations in human liver microsomes revealed no differences in CYP activities in adult and elderly subjects [42,43]. Our results show declines in the AUC ratio between adulthood and advanced age. This suggests that aging, considering previous studies reporting uncompromised CYP activity, reduces the inhibitory effect of fluvoxamine, as the AUC ratio value decreases (enhanced salbutamol metabolization). 

Additionally, we studied whether the previously reported tendency of increased salbutamol kinetics with an increasing fluvoxamine dosage in a 30-year-old patient would extend to the other age groups. Our results follow the same pattern (see Figure 5), with greater evidence in the older subject. Therefore, the concurrent administration of fluvoxamine and salbutamol should be under close observation in every age group in order to prevent possible adverse outcomes that could result from the increased plasma concentration of salbutamol. Along with this, clinicians may need to readjust the fluvoxamine dosage in patients taking salbutamol on a daily basis. Of note, prescribing more than 100 mg of fluvoxamine is contraindicated in children; thus, the other combinations cannot be extrapolated to human clinical trials.

### 3.5. Effect of Comorbidities on Salbutamol Pharmacokinetics Co-Administered with Fluvoxamine

Using different PBPK models based on weight and renal function, the interaction between the SABA and antidepressant was further investigated. Table 6 summarizes the systemic and hepatic AUC ratios of salbutamol in patients with different physiological statuses. In patients with excessive weight, the AUC ratio values are all slightly lower compared to an individual with a normal weight (healthy). These numbers are also reduced in cases of obesity. Therefore, our results go beyond previous studies. Indeed, obesity, as a metabolic disorder, is associated with disturbances in metabolism, leading to an increased risk of ADRs and DDIs. Tamankova et al. [44] have reviewed the effects of obesity on CYP properties. Specifically, the few studies published on the effect of obesity on CYP2D6 expression are contradictory. CYP2C19 activity, in turn, is reported to be higher in obese than in non-obese individuals [45]. Amplified CYP2C19 protein expression may explain our results. Recognizing that CYP2D6 is inhibited and CYP2C19 activity is not impacted by fluvoxamine intake, the increase in CYP2C19 may cover up the fluvoxamine’s inhibitory effect; hence, we observed reduced salbutamol AUC ratios in overweight individuals. Thus, we may conclude that increased weight weakens the salbutamol–fluvoxamine interaction.

Likewise, altered renal function, according to our simulations, does not significantly influence the salbutamol–fluvoxamine interaction, since the salbutamol AUC ratio does not vary considerably. Moreover, the severity of renal impairment has no impact on the administration of both drugs, as the AUC ratio are not differentiable. Déri et al. [46] aimed to compare the expression of CYP enzymes in patients with end-stage kidney disease and in healthy individuals. The results indicated a transcription down-regulation of CYP genes in patients with renal impairment, thereby compromising enzymatic activity. Thus, we may correlate kidney function with its transition from an extensive CYP-metabolizer to a poor CYP-metabolizer. The non-metabolization of the drug at its maximal rate would then explain the increased salbutamol AUC ratio. Additionally, fluvoxamine as a CYP2D6 inhibitor should potentiate the salbutamol plasma concentration when combined with it. In our study, we evidenced, conversely, a decrease in the AUC ratio in individuals with renal impairment, compared to the healthy ones. Remarkably, the aforementioned proportional increase in the AUC ratio throughout different treatment regimens (100, 200, and 300 mg of fluvoxamine) was observed as well.

The prescription of medication during pregnancy, especially in the first trimester, is associated with a high degree of uncertainty, due to the potential risks that some drugs might produce in the fetus and in the woman herself [47]. This critical risk derives from the fact that pregnancy alters the PK profile of several drugs, particularly in terms of hepatic metabolism [48]. The use of salbutamol monotherapy is not contraindicated in pregnancy, and fluvoxamine may be administered under medical supervision [49,50]. However, both drugs belong to the FDA pregnancy category C, which means that the risk of administering these drugs cannot be predicted, as there are still no satisfactory studies in pregnant women [47]. With this in mind, we attempted to determine whether the co-administration of these two drugs poses a risk to the pregnant woman and the fetus. Therefore, we used a specific PBPK model for a 30-year-old pregnant American woman and compared its AUC ratio with a non-pregnant 30-year-old American woman (Table 7). Our findings do not demonstrate the aforementioned trend. There are effectively no differences in the AUC ratio between non-pregnant and pregnant women, suggesting that the fluvoxamine–salbutamol interaction is not influenced by this condition. We may therefore conclude that this therapeutic regimen of salbutamol and fluvoxamine is safe in pregnant woman.

Some studies reveal incremental increases in CYP2A6, CYP2C9, CYP2D6, and CYP3A4 function in pregnant women, whereas those of CYP1A2 and CYP2C19 are decreased [48,51]. Despite the fact that mechanisms of altered CYP-mediated metabolism are not well described, it is believed that gestational hormones play an active role in regulating the expression of these proteins [48]. According to these statements, the prediction of salbutamol metabolism is challenging, since it is metabolized by increased CYP2D6 and decreased CYP2C19, respectively, during pregnancy. Given that metabolic rate is not remarkably affected, we may conclude that pregnancy has little impact on salbutamol regular intake. In fact, as we previously mentioned, salbutamol monotherapy is not contraindicated in pregnancy, suggesting that no ADRs are reported. Nevertheless, salbutamol drug exposure may be altered when co-administered with other medications. Thus, the interaction of fluvoxamine with salbutamol at the CYP2D6 inhibition level, together with reduced CYP2C19 in pregnancy, could potentiate the non-clearance of the parent drug, leading to increased serum levels. As a result, higher AUC ratios are expected in pregnant compared to non-pregnant women. Our results do not corroborate this theory. 

Although it was not a primary goal of this study, our results cast a new light on the relevance of gender as a covariate in the interaction of salbutamol with fluvoxamine, since we obtained substantially lower AUC ratios for women than for men. As a matter of fact, being a woman or a man has an impact on drug PKs, mainly due to sex-based differences in metabolism [52,53,54]. Women, for instance, exhibit greater CYP2D6 activity than men [55]. Regarding CYP2C19, there have been no reports of significant variations between both genders [54]. Having said that, the metabolic rate of salbutamol is higher due to the greater activity of CYP2D6, leading to a drop in the victim drug’s plasma levels. This explains the reduced AUC ratio values in women compared to men. Thus, the influence of fluvoxamine on salbutamol kinetics is not as evident in women as in men.

## 4. Conclusions

In silico studies of PK interaction between two drugs are scarce. Here, we developed for the first time a predictive model of the CYP-mediated interaction of salbutamol with an antidepressant drug, fluvoxamine. We have demonstrated the influence of several covariates, namely the age, renal function, weight, and pregnancy status. Furthermore, we have highlighted the need for monitoring when the dose of fluvoxamine is raised, due to the high risk of toxicity and unpleasant effects caused by an increase in the salbutamol plasma concentration. We are aware of the limitations of this study, though. Despite us assuming that salbutamol is exclusively metabolized by CYP enzymes, other metabolization pathways (sulfotransferases) that may influence the salbutamol–fluvoxamine interaction has been identified. Furthermore, we also disregarded the possibility that salbutamol itself may affect drug exposure by CYP2D6 inhibition. In vitro assays should be conducted to complement what we have presented here. Therefore, this study may be extrapolated to other medicines and serves as a pioneer for future PK DDI studies. As a take-home message, the prescription of various drugs must always be supervised, regardless of the patient’s characteristics, since this may result in non-accomplished therapeutic effects or undesirable consequences. 

## Figures and Tables

**Table 1:** Predicted and optimized physicochemical properties of the drug salbutamol.

Physicochemical Properties	Predicted Value	Optimized Value	Reference
Log P	1.644	1.4	[18,21,22,23,24]
Ionization constant (pKa)	9.98	10.3	
Molecular Weight (g/mol)	239.317	239.31	
Water Solubility (mg/mL)	15.869	9.53	[22]
Diff. Coeff. (cm^2^/s·10^5^)	0.804	ND	ND
Peff (cm/s·10^4^)	1.331	0.815	Calculated from pkCSM [21]
BBB penetration	Low	Low	Calculated from SwissADME and pkCSM [21,22]

Diff. Coeff, differential coefficient; Peff, effective human jejunal permeability; BBB, blood–brain barrier; ND, not defined.

**Table 2:** Metabolic profile of salbutamol and fluvoxamine.

Drug	CYP Enzyme	Inhibitor	Substrate	Km	V_max_	CL	Sites of Metabolism
Salbutamol	1A2	No (90%)	No (97%)	NS	NS	NS	NS
	2A6	ND	No (98%)	NS	NS	NS	NS
	2B6	ND	No (65%)	NS	NS	NS	NS
	2C8	ND	No (92%)	NS	NS	NS	NS
	2C9	No (99%)	No (98%)	NS	NS	NS	NS
	2C19	ND	Yes (82%)	30.146	157.577	73.179	C7
	2D6	Yes (49%)	Yes (66%)	37.808	2.201	0.466	C17
	2E1	ND	No (91%)	NS	NS	NS	NS
	3A4	No (78%)	No (84%)	NS	NS	NS	NS
Fluvoxamine	1A2	No (51%)	Yes (48%)	1.821	1.500	42.835	C1, C11
	2A6	ND	No (82%)	NS	NS	NS	NS
	2B6	ND	No (83%)	NS	NS	NS	NS
	2C8	ND	No (99%)	NS	NS	NS	NS
	2C9	Yes (41%)	No (78%)	NS	NS	NS	NS
	2C19	No (95%)	Yes (67%)	22.656	250.097	154.542	C1, C3, C11, C12
	2D6	Yes (70%)	Yes (66%)	0.674	3.937	46.721	C1, C3, C11
	2E1	ND	Yes (78%)	ND	ND	ND	C1, C3, C12
	3A4	Yes (80%)	No (54%)	NS	NS	NS	NS

ND, not defined; NS, no substrate.

**Table 3:** Observed (ADMET Predictor^®^) and estimated (GastroPlus) pharmacokinetic properties of 4 mg salbutamol administered every 6 h after a 24 h simulation.

Pharmacokinetic Parameters	Observed Value	Estimated Value
Fa (%)	88.82	88.079
FDp (%)	ND	87.486
F (%)	ND	29.447
C_max_ (μg/mL)	0.01013	3.159 × 10^−3^
T_max_ (h)	2.73	19.84
AUC_0–inf_ (μg*h/mL)	0.1094	0.05235
AUC_0–t_ (μg*h/mL)	0.1094	0.04929
C_max liver_ (μg/mL)	ND	7.57 × 10^−3^

ND, not defined.

**Table 4:** Effect of increasing fluvoxamine dose on the pharmacokinetics of salbutamol. Pharmacokinetic parameters were estimated by dynamic simulation for 24 h in a 30-year-old American male undergoing fixed-dose salbutamol (4 mg q6h) and SID fluvoxamine therapy.

Compound	Fa (%)	FDp (%)	F (%)	C_max_ (μg/mL)	T_max_ (h)	AUC_0–t_ (ng.h/mL)	AUC_0-inf_ (ng.h/mL)
Salbutamol baseline	88.08	87.49	29.44	0.0032	19.76	52.34	49.65
Salbutamol 4 mg + fluvoxamine 100 mg	88.06	87.47	38.50	0.0052	7.92	78.04	74.19
Salbutamol 4 mg + fluvoxamine 200 mg	88.04	87.45	44.76	0.0067	7.92	100.6	95.39
Salbutamol 4 mg + fluvoxamine 300 mg	88.03	87.43	49.41	0.0080	8.00	120.6	113.9

**Table 5:** Interaction of different doses (100, 200, and 300 mg) of fluvoxamine on the pharmacokinetics of salbutamol (4 mg q6h) in 10-, 30-, and 65-year-old virtual subjects.

Dosing Regimen	Concentration Type	AUC Ratio			DDI Classification		
Age		10	30	65	10	30	65
Salbutamol with fluvoxamine 100 mg	C_max_	3.453	3.630	3.431	M	M	M
	Liver Unbound	2.726	2.021	2.586	M	M	M
Salbutamol with fluvoxamine 200 mg	C_max_	3.854	3.973	3.839	M	M	M
	Liver Unbound	3.287	2.558	3.156	M	M	M
Salbutamol with fluvoxamine 300 mg	C_max_	4.022	4.111	4.011	M	M	M
	Liver Unbound	3.567	2.903	3.456	M	M	M

M, moderate interaction.

**Table 6:** Interaction of different doses (100, 200, and 300 mg) of fluvoxamine on the pharmacokinetics of salbutamol (4 mg q6h) in a 30-year-old American male with different physiological status.

Dosing Regimen	Concentration Type	AUC Ratio						DDI Classification					
Physiological Status		Healthy	OW	Obese	MildRI	MRI	SRI	Healthy	OW	Obese	MildRI	MRI	SRI
Salbutamol 4 mg + fluvoxamine 100 mg	C_max_	3.630	3.420	3.413	3.423	3.423	3.423	M	M	M	M	M	M
	Liver Unbound	2.021	2.501	2.451	2.511	2.511	2.511	M	M	M	M	M	M
Salbutamol 4 mg + fluvoxamine 200 mg	C_max_	3.973	3.831	3.827	3.832	3.832	3.832	M	M	M	M	M	M
	Liver Unbound	2.558	3.075	3.031	3.089	3.089	3.089	M	M	M	M	M	M
Salbutamol 4 mg + fluvoxamine 300 mg	C_max_	4.111	4.005	4.001	4.006	4.006	4.006	M	M	M	M	M	M
	Liver Unbound	2.903	3.385	3.341	3.396	3.396	3.396	M	M	M	M	M	M

OW, overweight; MildRI, mild renal impairment; MRI, moderate renal impairment; SRI, severe renal impairment, M, moderate interaction.

**Table 7:** Interaction of different doses (100, 200, and 300 mg) of fluvoxamine on the PK of salbutamol (4 mg q6h) in a 30-year-old pregnant woman.

Dosing Regimen	Concentration Type	AUC Ratio		DDI Classification	
Age		Female	Pregnant	Female	Pregnant
Salbutamol 4 mg + fluvoxamine 100 mg	C_max_	3.426	3.426	M	M
	Liver Unbound	2.530	2.540	M	M
Salbutamol 4 mg + fluvoxamine 200 mg	C_max_	3.835	3.835	M	M
	Liver Unbound	3.112	3.117	M	M
Salbutamol 4 mg + fluvoxamine 300 mg	C_max_	4.007	4.007	M	M
	Liver Unbound	3.415	3.420	M	M

M, Moderate interaction.

## Data Availability

Not applicable.

## Supplementary Materials

The following supporting information can be downloaded at: https://www.mdpi.com/article/10.3390/pharmaceutics15061586/s1, Table S1: List of potential salbutamol-interacting drugs, belonging to different drug classes, from corticosteroids to antidepressants; Table S2: PBPK models and respective individuals’ characteristics.; Table S3: Metabolic profile of the 17 potential salbutamol-interacting drugs, estimated by ADMET Predictor^®^.

## Abbreviations

Drug–drug interactions (DDIs), adverse reactions (ADRs), pharmacokinetic (PK), pharmacodynamic (PD), cytochrome (CYP), maximum concentration (C_max_), area under curve (AUC), Global Initiative for Asthma (GINA), inhaled corticosteroids (ICS), physiologically based pharmacokinetic (PBPK), human jejunum effective permeability (Peff), diffusion coefficient (Diff.Coeff.), blood–brain barrier (BBB), fraction absorbed (Fa), fraction of the drug concentration in the portal vein (FDp), fraction of the drug concentration in blood (F), maximum concentration in liver (C_max liver_), time required to maximum plasma concentration (T_max_), absorption, distribution, metabolism, excretion, and toxicity (ADMET), Body Mass Index (BMI), estimated glomerular filtration rate (eGFR), kinetic constant (Ki), half-maximal inhibitory concentration (IC_50_), half-maximal induction concentration (EC_50_), ionization constant (pKa), Michaelis–Menten constant (Km), maximum rate of metabolization (V_max_), intrinsic clearance (CL), once daily (SID), overweight (OW), mild renal impairment (MildRI), moderate renal impairment (MRI), severe renal impairment (SRI).
