# Supplementary material for: Prediction of CYP-Mediated Drug Interaction Using Physiologically Based Pharmacokinetic Modeling: A Case Study of Salbutamol and Fluvoxamine
Source: Pharmaceutics. 2023 May 24;15(6):1586. doi: 10.3390/pharmaceutics15061586 (PMC10301358; doi:10.3390/pharmaceutics15061586)
Supplement: Supplementary file 1 [file pharmaceutics-15-01586-s001.zip › pharmaceutics-2390846-supplementary.pdf]

Supplementary Materials: Prediction of CYP-mediated drug interaction using physiologically based pharmacokinetic modeling: A case study of salbutamol and fluvoxamine

Lara Marques [1,2,3] and Nuno Vale [1,3,4,*]

[1] OncoPharma Research Group, Center for Health Technology and Services Research (CINTESIS), Rua Doutor Plácido da Costa, 4200-450 Porto, Portugal
[2] Faculty of Medicine, University of Coimbra, Azinhaga de Santa Comba, Celas, 3000-548 Coimbra, Portugal
[3] CINTESIS@RISE, Faculty of Medicine, University of Porto, Al. Prof. Hernâni Monteiro, 4200-319 Porto, Portugal
[4] Department of Community Medicine, Health Information and Decision (MEDCIDS), Faculty of Medicine, University of Porto, Rua Dr. Plácido da Costa, 4200-450 Porto, Portugal
* Correspondence: nunovale@med.up.pt; Tel.: +351-220426537

Table S1. List of potential salbutamol-interacting drugs, belonging to different drug classes, from corticosteroids to antidepressants.

**Drug**	**Drug Class**
Atenolol	β-blocker
Beclomethasone	Corticosteroid
Doxepin	Tricyclic antidepressant
Fluticasone Propionate	Corticosteroid
Fluticasone	Corticosteroid
Formoterol	Long-acting β-agonist
Furosemide	Diuretic
Isocarboxazid	Monoamine oxidase inhibitor
Ketotifen	Antihistamine
Methylprednisolone	Corticosteroid
Prednisolone	Corticosteroid
Prednisone	Corticosteroid
Propranolol	β-blocker
Rosiglitazone	Thiazolidinedione
Salmeterol	Long-acting β-agonist
Theophylline	Xanthine
Fluvoxamine	Selective serotonin reuptake inhibitor

Table S2. PBPK models and respective individuals' characteristics.

PBPK Model	Population	Gender	Age (years)	Health Status	Height (cm)	Weight (kg)	BMI (kg/m²)	Body Fat (%)
1	American	Male	30	Healthy	176.43	75.0	24.0944	23.41
2	American	Male	10	Healthy	145.23	38.9	18.4432	17.18
3	American	Male	65	Healthy	173.76	75.0	24.8406	24.08
4	American	Male	30	Mild Renal Impairment	176.43	75.0	24.0944	23.41
5	American	Male	30	Moderate Renal Impairment	176.43	75.0	24.0944	23.41
6	American	Male	30	Severe Renal Impairment	176.43	75.0	24.0944	23.41
7	American	Male	30	Healthy (Overweight)	176.43	85.53	27.4773	26.34
8	American	Male	30	Healthy (Obese)	176.43	105.0	33.7322	31.80
9	American	Female	30	Healthy	162.20	65.0	24.71	33.28
10	American	Female	30	Healthy (Pregnant)	162.20	65.0	24.71	33.28

BMI, Body Mass Index

Table S3. Metabolic profile of the 17 potential salbutamol-interacting drugs, estimated by ADMET Predictor ®.

Drug	CYP Enzyme	Inhibitor	Substrate	Km	V_{max}	CL	Sites of metabolism
Atenolol	1A2	No (63%)	No (90%)	NS	NS	NS	NS
	2A6	ND	No (98%)	NS	NS	NS	NS
	2B6	ND	No (98%)	NS	NS	NS	NS
	2C8	ND	No (92%)	NS	NS	NS	NS
	2C9	No (95%)	Yes (98%)	NS	NS	NS	NS
	2C19	No (99%)	Yes (39%)	74.179	422.087	79.662	C17, C7
	2D6	Yes (46%)	Yes (66%)	0.778	1.291	13.282	C17, C7
	2E1	ND	No (98%)	NS	NS	NS	NS
	3A4	No (78%)	No (84%)	NS	NS	NS	NS
Beclomethasone	1A2	No (82%)	No (97%)	NS	NS	NS	NS
	2A6	ND	No (98%)	NS	NS	NS	NS
	2B6	ND	Yes (71%)	ND	ND	ND	C28, C34 e C27
	2C8	ND	No (63%)	NS	NS	NS	NS
	2C9	No (99%)	No (98%)	NS	NS	NS	NS
	2C19	No (95%)	No (88%)	74.179	422.087	79.662	C17, C7
	2D6	No (95%)	No (95%)	0.778	1.291	13.282	C17, C7
	2E1	ND	No (84%)	NS	NS	NS	NS
	3A4	Yes (80%)	Yes (89%)	40.448	16.885	46.338	C7, C33 e C27

Doxepin	1A2	No (97%)	Yes (91%)	23.920	10.658	23.171	C20 e C21
	2A6	ND	No (70%)	NS	NS	NS	NS
	2B6	ND	Yes (89%)	ND	ND	ND	C20, C21 e C11
	2C8	ND	Yes (91%)	ND	ND	ND	C20, C21
	2C9	No (90%)	Yes (66%)	5.823	0.149	1.867	C20, C21
	2C19	Yes (31%)	Yes (82%)	69.614	15.082	3.033	C20, C21
	2D6	Yes (51%)	Yes (87%)	3.417	7.545	17.667	C20, C21, C13 e C11
	2E1	ND	No (69%)	NS	NS	NS	NS
	3A4	No (90%)	Yes (74%)	74.034	4.391	6.584	C20, C21
Fluticasone Propionate	1A2	No (76%)	No (97%)	NS	NS	NS	NS
	2A6	ND	No (98%)	NS	NS	NS	NS
	2B6	ND	Yes (44%)	ND	ND	ND	S28
	2C8	ND	No (86%)	NS	NS	NS	NS
	2C9	No (99%)	No (88%)	NS	NS	NS	NS
	2C19	No (89%)	No (88%)	NS	NS	NS	NS
	2D6	No (95%)	No (85%)	NS	NS	NS	NS
	2E1	ND	No (76%)	NS	NS	NS	NS
	3A4	Yes (80%)	Yes (98%)	19.997	6.993	39.033	S28, C7, C33 e C29
Fluticasone	1A2	No (90%)	No (97%)	NS	NS	NS	NS
	2A6	ND	No (98%)	NS	NS	NS	NS
	2B6	ND	Yes (61%)	ND	ND	ND	S28
	2C8	ND	Yes (58%)	ND	ND	ND	C29, S28, C16, C24, C19 e C7
	2C9	No (99%)	No (96%)	NS	NS	NS	NS
	2C19	No (94%)	No (88%)	NS	NS	NS	NS
	2D6	No (95%)	No (95%)	NS	NS	NS	NS
	2E1	ND	No (71%)	NS	NS	NS	NS
	3A4	Yes (38%)	Yes (98%)	40.068	1.515	4.197	S28, C7, C29
Formoterol	1A2	Yes (58%)	No (97%)	NS	NS	NS	NS
	2A6	ND	No (94%)	NS	NS	NS	NS
	2B6	ND	No (98%)	NS	NS	NS	NS
	2C8	ND	No (92%)	NS	NS	NS	NS
	2C9	No (95%)	No (98%)	NS	NS	NS	NS
	2C19	No (96%)	No (88%)	NS	NS	NS	NS
	2D6	Yes (70%)	Yes (87%)	5.614	8.237	11.739	C19, C23
	2E1	ND	No (98%)	NS	NS	NS	NS
	3A4	No (46%)	No (48%)	NS	NS	NS	NS
Furosemide	1A2	No (86%)	No (97%)	NS	NS	NS	NS
	2A6	ND	No (70%)	NS	NS	NS	NS
	2B6	ND	No (98%)	NS	NS	NS	NS
	2C8	ND	No (77%)	NS	NS	NS	NS
	2C9	Yes (49%)	Yes (45%)	5.540	3.661	48.237	C2 e C6
	2C19	Yes (99%)	Yes (82%)	1813.378	3637.938	38.086	C6, C2 e C1
	2D6	No (55%)	No (68%)	NS	NS	NS	NS
	2E1	ND	Yes (82%)	ND	ND	ND	C2
	3A4	No (80%)	No (38%)	NS	NS	NS	NS
Isocarboxazid	1A2	Yes (95%)	Yes (74%)	524.501	15.319	1.519	C7, C17 e C3
	2A6	ND	Yes (82%)	ND	ND	ND	C7, C17, N13 e C3
	2B6	ND	No (89%)	NS	NS	NS	NS
	2C8	ND	Yes (58%)	ND	ND	ND	C7, C17 e C3
	2C9	Yes (41%)	No (88%)	NS	NS	NS	NS
	2C19	No (78%)	Yes (61%)	57.052	132.576	32.533	C17 e C7
	2D6	No (95%)	No (68%)	NS	NS	NS	NS
	2E1	ND	Yes (40%)	ND	ND	ND	C7, C3 e N13
	3A4	Yes (51%)	No (58%)	NS	NS	NS	NS

Ketotifen	1A2	No (90%)	Yes (91%)	139.473	9.355	3.488	C22 e N19
	2A6	ND	Yes (ND)	ND	ND	ND	C22 e C12
	2B6	ND	No (ND)	NS	NS	NS	NS
	2C8	ND	No (77%)	NS	NS	NS	NS
	2C9	No (ND)	Yes (ND)	21.787	0.107	0.359	C22
	2C19	Yes (ND)	Yes (ND)	50.763	5.845	1.612	C22
	2D6	Yes (55%)	Yes (ND)	21.857	27.720	10.146	C22, C12, C7, C3 e N19
	2E1	ND	No (76%)	NS	NS	NS	NS
	3A4	No (90%)	Yes (92%)	53.902	4.131	8.507	C22, N19 e C12
Methylprednisolone	1A2	No (97%)	No (97%)	NS	NS	NS	NS
	2A6	ND	No (98)	NS	NS	NS	NS
	2B6	ND	Yes (47%)	ND	ND	ND	C25
	2C8	ND	No (66%)	NS	NS	NS	NS
	2C9	No (99%)	No (98%)	NS	NS	NS	NS
	2C19	No (95%)	No (99%)	NS	NS	NS	NS
	2D6	No (95%)	No (95%)	NS	NS	NS	NS
	2E1	ND	No (78%)	NS	NS	NS	NS
	3A4	Yes (46%)	Yes (98%)	92.176	37.806	45.527	C7, C25 e C13
Prednisolone	1A2	No (97%)	No (97%)	NS	NS	NS	NS
	2A6	ND	No (98%)	NS	NS	NS	NS
	2B6	ND	Yes (46%)	ND	ND	ND	C24 e C12
	2C8	ND	No (77%)	NS	NS	NS	NS
	2C9	No (99%)	No (98%)	NS	NS	NS	NS
	2C19	No (97%)	No (95%)	NS	NS	NS	NS
	2D6	No (95%)	No (95%)	NS	NS	NS	NS
	2E1	ND	No (80%)	NS	NS	NS	NS
	3A4	Yes (38%)	Yes (92%)	83.372	24.021	31.981	C7, C24 e C12
Prednisone	1A2	No (97%)	No (90%)	NS	NS	NS	NS
	2A6	ND	No (98%)	NS	NS	NS	NS
	2B6	ND	Yes (49%)	ND	ND	ND	C23
	2C8	ND	No (80%)	NS	NS	NS	NS
	2C9	No (99%)	No (98%)	NS	NS	NS	NS
	2C19	No (98%)	No (95%)	NS	NS	NS	NS
	2D6	No (95%)	No (95%)	NS	NS	NS	NS
	2E1	ND	No (84%)	NS	NS	NS	NS
	3A4	Yes (46%)	Yes (98%)	74.801	11.076	16.436	C7, C23
Propanolol	1A2	No (61%)	No (97%)	NS	NS	NS	NS
	2A6	ND	No (98%)	NS	NS	NS	NS
	2B6	ND	No (73%)	NS	NS	NS	NS
	2C8	ND	No (92%)	NS	NS	NS	NS
	2C9	No (92%)	No (98%)	NS	NS	NS	NS
	2C19	No (65%)	No (99%)	NS	NS	NS	NS
	2D6	No (55%)	Yes (87%)	3.901	1.458	2.990	C17
	2E1	ND	No (98%)	NS	NS	NS	NS
	3A4	No (90%)	No (58%)	NS	NS	NS	NS
Rosiglitazone	1A2	No (90%)	No (91%)	NS	NS	NS	NS
	2A6	ND	No (63%)	NS	NS	NS	NS
	2B6	ND	No (78%)	NS	NS	NS	NS
	2C8	ND	Yes (83%)	ND	ND	ND	C23 e C4
	2C9	Yes (63%)	Yes (66%)	117.900	8.272	5.122	S19, C23, C4 e C5
	2C19	Yes (23%)	Yes (82%)	15.478	0.964	0.872	C23, S19 e C9
	2D6	No (95%)	Yes (56%)	9.274	11.191	9.654	S19, C4 e C23
	2E1	ND	Yes (70%)	ND	ND	ND	S19, C4 e C23
	3A4	No (67%)	Yes (82%)	35.382	4.836	15.171	C23 e S19
Salmeterol	1A2	Yes (72%)	No (97%)	NS	NS	NS	NS

	2A6	ND	No (94%)	NS	NS	NS	NS
	2B6	ND	No (83%)	NS	NS	NS	NS
	2C8	ND	No (99%)	NS	NS	NS	NS
	2C9	No (90%)	No (90%)	NS	NS	NS	NS
	2C19	No (91%)	No (72%)	NS	NS	NS	NS
	2D6	Yes (70%)	Yes (72%)	3.054	37.871	99.216	C7, C20, C11, C23 e C27
	2E1	ND	No (82%)	NS	NS	NS	NS
	3A4	Yes (51%)	No (51%)	NS	NS	NS	NS
Theophylline	1A2	No (86%)	Yes (91%)	173.268	1.901	0.570	C12 e C7
	2A6	ND	Yes (82%)	ND	ND	ND	C12
	2B6	ND	No (75%)	NS	NS	NS	NS
	2C8	ND	No (99%)	NS	NS	NS	NS
	2C9	No (99%)	No (72%)	NS	NS	NS	NS
	2C19	No (98%)	No (95%)	NS	NS	NS	NS
	2D6	No (95%)	No (95%)	3.054	37.871	99.216	C7, C20, C11, C23 e C27
	2E1	ND	Yes (49%)	ND	ND	ND	C7, C10 e C12
	3A4	No (81%)	No (38%)	NS	NS	NS	NS

ND, not defined; NS, no substrate
